# Atelectasis in obese patients undergoing laparoscopic bariatric surgery are not increased upon discharge from Post Anesthesia Care Unit

**DOI:** 10.3389/fmed.2023.1233609

**Published:** 2023-08-29

**Authors:** Matthias Braun, Lea Ruscher, Alexander Fuchs, Martina Kämpfer, Markus Huber, Markus M. Luedi, Thomas Riva, Andreas Vogt, Thomas Riedel

**Affiliations:** ^1^Department of Anaesthesiology, Lindenhof Hospital, Bern, Switzerland; ^2^Department of Anaesthesiology and Pain Medicine, Bern University Hospital, Inselspital, University of Bern, Bern, Switzerland; ^3^Unit for Research in Anaesthesia, IRCCS Istituto Giannina Gaslini, Genoa, Italy; ^4^Division of Paediatric Intensive Care Medicine, Department of Paediatrics, Inselspital, Bern University Hospital, University of Bern, Bern, Switzerland

**Keywords:** adipositas, general anesthesia, laparoscopic surgery, bariatric (weight loss) surgery, mechanical ventilation

## Abstract

**Background:**

Obese patients frequently develop pulmonary atelectasis upon general anesthesia. The risk is increased during laparoscopic surgery. This prospective, observational single-center study evaluated atelectasis dynamics using Electric Impedance Tomography (EIT) in patients undergoing laparoscopic bariatric surgery.

**Methods:**

We included adult patients with ASA physical status I–IV and a BMI of ≥40. Exclusion criteria were known severe pulmonary hypertension, home oxygen therapy, heart failure, and recent pulmonary infections. The primary outcome was the proportion of poorly ventilated lung regions (low tidal variation areas) and the global inhomogeneity (GI) index assessed by EIT before discharge from the Post Anesthesia Care Unit compared to these same measures prior to initiation of anesthesia.

**Results:**

The median (IQR) proportion of low tidal variation areas at the different analysis points were T1 10.8% [3.6–15.1%] and T5 10.3% [2.6–18.9%], and the mean difference was −0.7% (95% CI: −5.8% −4.5%), i.e., lower than the predefined non-inferiority margin of 5% (*p* = 0.022). There were no changes at the four additional time points compared to T1 or postoperative pulmonary complications during the 14 days following the procedure.

**Conclusion:**

We found that obese patients undergoing laparoscopic bariatric surgery do not leave the Post Anesthesia Care Unit with increased low tidal variation areas compared to the preoperative period.

## Introduction

Postoperative pulmonary complications (PPC) increase morbidity, mortality, length of hospital stay, and costs (1–3). A frequent PPC is the formation of atelectasis, which is increased under general anesthesia, supine position, and controlled ventilation. Increased intra-abdominal pressure in laparoscopic surgery also increases the risk of atelectasis formation (1). Obese patients are more affected by developing postoperative pulmonary complications and atelectasis under general anesthesia (1, 4, 5). They tend to take longer to reopen atelectasis than non-obese patients and experience poor ventilation over a significant period of time (6, 7).

Protective ventilatory strategies using lower tidal volumes, increased positive end-expiratory pressure (PEEP), and recruitment maneuvers (8, 9) have been shown to reduce PPC, including atelectasis, and to result in a shorter postoperative anesthesia care unit (PACU) stay (1, 10). The combination of recruitment maneuvers and PEEP could lead to transiently improved oxygenation (4). Nevertheless, obese patients often receive high tidal ventilation with low PEEP and rarely receive recruitment maneuvers (11).

Intraoperative alveolar recruitment followed by PEEP of 10 cm H_2_O was associated with less atelectasis assessed with computer tomography compared to no PEEP or PEEP of 5 cm H_2_O in obese patients undergoing laparoscopic bariatric surgery (1).

In a cohort of obese patients, 10 cm H_2_O PEEP improved oxygenation but did not reverse the formation of atelectasis intraoperatively (12). Individually titrated PEEP by electrical impedance tomography (EIT) and recruitment maneuvers lead to pulmonary conditions being on a level comparable to preoperative conditions in obese patients. Nevertheless, atelectasis was reformed before discharge from the PACU, and end-expiratory lung volume was lower than the preoperative value (13). On the contrary, in small children, a population physiologically similar to obese patients, general anesthesia maintaining spontaneous ventilation homogeneity was fully resolved at discharge from the PACU (14).

Actual bariatric surgery numbers are rising fast, and more obese patients receive surgery. With the persisting trend for ambulatory or short-stay surgery, it is essential to know which pulmonary condition patients can be discharged early from PACU and the hospital to avoid costly hospital stays and readmission. However, the potential benefits of 10 cm H_2_0 PEEP and repeated recruitment maneuvers to prevent perioperative atelectasis in this cohort are unclear. We aimed to investigate perioperatively atelectasis formation in obese patients scheduled for laparoscopic bariatric surgery from the preoperative phase until discharge from the PACU and the hospital, using electrical impedance tomography: a technique that can detect minimally ventilated lung regions rapidly, continuously, and without harmful radiation or other physical harm to the patient.

## Materials and methods

The study was approved by the local ethics committee of the Canton of Bern (BASEC 2021-01473) and prospectively registered with ClinicalTrials.gov (NCT05187039). Written informed consent was obtained from each participant.

### Study design and patients

We included 30 patients in this single-center prospective observational trial conducted at the Department of Anesthesiology and Pain Medicine at Bern University Hospital, Switzerland, from 01 November 2021 to 30 September 2022.

The inclusion criteria were patients over 18 years of age who provided written informed consent and were scheduled for laparoscopic bariatric surgery with a body mass index (BMI) of ≥40 and an ASA physical status of I to IV.

Exclusion criteria were known severe pulmonary hypertension, need for home oxygen therapy, known heart failure, and suspected or known recent pulmonary infections. Eligible patients were screened from the preadmission clinic or operating room lists and recruited in the preadmission clinic or the preadmission area.

**Figure 1:**
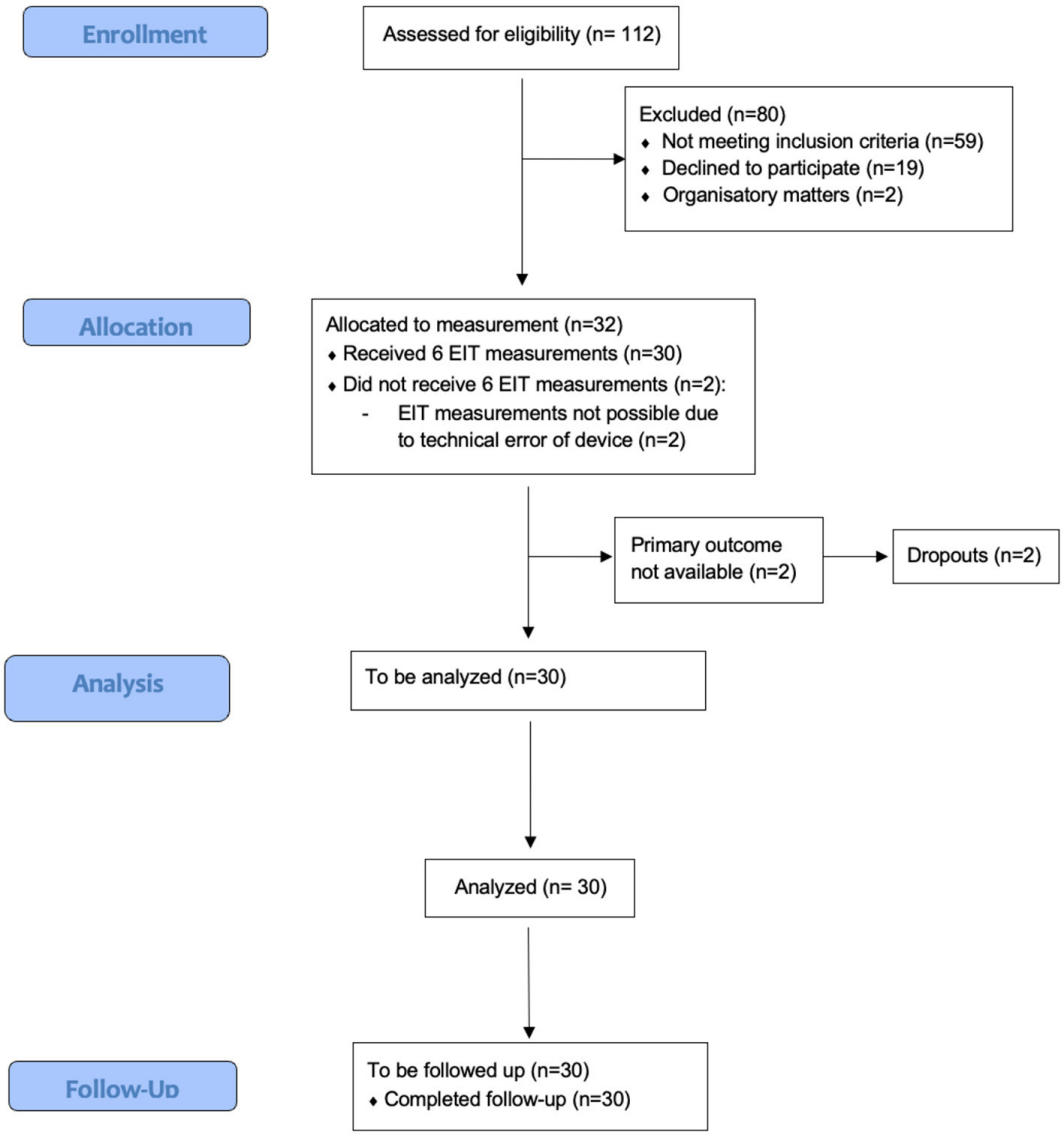
STROBE diagram.

All methods were performed in accordance with the relevant guidelines and regulations and this manuscript follows the applicable STROBE guidelines (see Figure 1).

### Anesthesia and measurement

The patients did not receive pharmacological premedication. Participating patients were prepared for general anesthesia according to the departmental standard operating procedure with SpO_2_, electrocardiogram, non-invasive blood pressure, quantitative neuromuscular monitoring (TOF-Watch, Organon Ltd, Dublin, Ireland), and an intravenous line. Anesthesia was induced with Propofol 2–3 mg/kg (predicted body weight), Fentanyl 2 mcg/kg (predicted body weight), and Succinylcholine 1 mg/kg (total body weight) or Rocuronium 0.9 mg/kg (predicted body weight) if Succinylcholine was contraindicated. The patients were induced with a modified rapid sequence intubation technique and were all ventilated after apnea started with bag-mask ventilation and a ventilatory pression not exceeding 10 mm Hg. Male participants were intubated with a cuffed tracheal tube size of 8.0 and female participants with 7.0. Successful tracheal intubation was confirmed with waveform capnography. Anesthesia was maintained by volatile anesthetics (MAC 0.75) and Fentanyl guided by an electroencephalogram (Narcotrend, Hannover, Germany). Rocuronium boluses were given to support a “train of four” ratio of 0 of 4. Co-analgesics (i.e., Dexmedetomidine and Ketamine) and other medications (e.g., Dexamethasone and Ondansetron) were given according to standard operating procedures in our bariatric surgery clinic. After intubation and before surgical incision, regional anesthesia (subcostal transversus abdominis plane block) was set using ultrasound guidance with 75 mg Ropivacaine 0.375% per side. While the patient was prepared for the surgical procedure and sterile drapes were applied, a standard recruitment maneuver (40 cmH_2_O for 10 s, repeated twice) was performed before surgical disinfection. Patients were ventilated with an anesthetic respirator (Primus, Draeger, Germany) in a volume-controlled mode with a tidal volume of 6–8 ml/kg ideal body weight in a frequency of 12–16/min, the PEEP level set at 10 cm H_2_O, and a fraction of inspired oxygen (FiO_2_) at 0.6. According to the capnography reading, the frequency was adjusted during pneumoperitoneum to maintain an end-tidal CO_2_ between 30 and 40 mmHg according to the usual standards of care. Anesthesia was usually induced in a ramped position by arranging a ramping cushion under the patient's upper body and head. Patients are positioned in an anti-Trendelenburg position shortly before the surgery started.

Before extubation, neuromuscular blocking was antagonized in all patients with 200 mg of Sugammadex to achieve a “train of four” ratio of >90%. During the emergence of anesthesia, the patients were ventilated in pressure support mode with a PEEP of 5 mm Hg and pressure support of 5–8 mm Hg. In PACU, all patients received conventional oxygen therapy with flows 4–6 l/min.

Thoracic electrical impedance tomography measurements (Pulmovista, Dräger, Germany) were performed at the following time points:

- T1: before induction of the anesthesia.
- T2: after intubation and recruitment maneuvers, before the surgical procedure.
- T3: after the surgical procedure, before extubation.
- T4: after extubation, before transfer to the PACU.
- T5: after 2 h of surveillance, before discharge from the PACU.
- T6: before discharge to home.

A loose-fitting belt with 16 evenly spaced electrodes was placed around each patient's chest between the 4 and 6th intercostal space in a thoracic median plane as soon as the patient arrived in the operation theater. The belt was removed after the T5-measurement before the patient was discharged from the PACU and transferred to the regular surgical ward. To ensure that the belt was reapplied in the same position before the T6-measurement, the upper and lower edges of the belt and the placement of the electrodes on the chest were labeled with a marker, which was renewed daily by ward nurses. Each measurement lasted 1 min. The EIT images were reconstructed based on the Graz consensus reconstruction algorithm for EIT (GREIT) using the torso mesh function based on generic CT scans (15, 16). No additional regions of interest were applied for any of the analyses. The following EIT parameters were calculated for each time point: percentage in low tidal variation areas (defined as areas within the thorax that exhibit <10% of the maximum detected tidal impedance change) and the global inhomogeneity (GI) index, a measure of ventilation inhomogeneity (17). All analyses used a custom code (MATLAB R2021a; The MathWorks Inc., Natick, MA, USA) (18–20). We performed a telephone follow-up 14 days after the surgery to inquire about postoperative pulmonary complications or the need for re-hospitalization.

Postoperative pulmonary complications were defined as respiratory failure, acute respiratory distress syndrome requiring reintubation, respiratory support or rehospitalization, bronchospasm, and new pulmonary infiltrates.

### Primary and secondary outcomes

The primary outcome was the percentage of low tidal variation areas derived from EIT before the discharge from PACU (T5) compared to the measurement before induction (T1).

This analysis is based on the analysis of Ukere et al. with the difference that we do not use anatomically defined lung regions (21). This has the advantage that no minimally ventilated lung regions are *a-priori* excluded from the analysis. The disadvantage is that areas outside the patient's lungs can also be included, which overestimates the low tidal variation areas. Secondary outcomes included changes from baseline (T1) in low tidal variation areas, ventilation inhomogeneity at all other time points (T2, T3, T4, and T6), and postoperative pulmonary complications until 14 days after the procedure.

### Statistical analysis

Currently, there are no data regarding the extent of atelectasis formation in patients undergoing bariatric surgery at the time of discharge from PACU. We hypothesized that the absolute percentage of low tidal variation areas at PACU discharge is at most 5% higher than before the operation, based on a small pilot study (*N* = 5), where the percentage of low tidal variation areas was measured before intubation and after PACU discharge. To investigate this hypothesis, we chose a non-inferiority design with a corresponding non-inferiority margin of 5% (absolute percentage). Formally, our hypothesis for the percentages of low tidal variation areas before the operation (π_pre_) and after PACU discharge (π_post_) can be stated as:

- Null hypothesis (H_0_): π_post_ > π_pre_ + Δ,
- Alternative hypothesis (H_1_): π_post_ ≤ π_pre_ + Δ,

where Δ refers to the non-inferiority margin of 5%.

Using the observed values of the pilot study (*N* = 5), we performed a simulation study to estimate the required sample size. Given the repeated measure design of the study and the continuous, bounded primary outcome (percentage of low tidal variation areas), the simulation was based on a generalized linear mixed model (GLMM) with a beta distribution for the outcome, resulting in an estimate of *N* = 20. So far, there are only data for the functional residual capacity and ventilation homogeneity impairment in anesthetized children exposed to high levels of inspired oxygen (22). This previous study set the sample size at 23 participants in each group. A similar study in adults set the sample size at 20 participants per group (23). Accounting for the pilot study's small size and possible dropouts, we envisaged a sample size of *N* = 30 patients. The initial sample size estimate could not be reproduced and a *post-hoc* validity check resulted in a power of 94.2% to declare non-inferiority (significance level of α = 0.025 and Δ = 5%) for *N* = 30 patients.

The primary outcome [change in the percentage of low tidal variation areas after PACU discharge (T5) relative to the percentage of low tidal variation areas before the operation (T1)] was analyzed in a regression framework: GLMM with a beta distribution was used to estimate the primary outcome. The GLMM was used to estimate a 95% confidence interval (CI) of the change in the percentage of low tidal variation areas, and the lower boundary of the CI will be compared to the non-inferiority margin. Non-inferiority—and the rejection of the Null hypothesis—will be declared if the lower boundary of 95% CI is smaller than the pre-defined non-inferiority margin of Δ (5%). The GLMM also allows controlling for covariates that could affect the perioperative reduction of lung impedance. As covariates, we include age and anesthesia time.

A secondary and explorative analysis examined *post-hoc* comparisons of the change from baseline (T1) in the percentage of low tidal variation areas and ventilation inhomogeneity at different time points (T2, T3, T4, and T6). For the secondary analyses, a Friedman Test was performed, and the corresponding contrasts were adjusted for multiple comparisons using the Conover method (24).

A *p* < 0.05 indicated statistical significance, and all analyses were performed in StatsDirect (StatsDirect Ltd, Wirral, United Kingdom) and R (25).

### Data availability

The datasets used and/or analyzed during the current study are available from the corresponding author upon reasonable request.

## Results

We recruited 32 patients, of which 30, for whom we obtained all measurements and follow-up, were analyzed (Figure 1). Due to a technical error of the EIT device, measurements could not be performed in two patients. Table 1 summarizes the baseline characteristics of the patients. The median age was 43 [32.5–48.0], and 19 patients (63.3%) were female. Median BMI was 45.6 [42.4–49.7] kg.m^−2^, and most patients had an ASA physical status of III (83.3%). Twenty-four patients obtained a laparoscopic gastric sleeve, while six obtained a laparoscopic gastric bypass. The mean duration of the surgical procedure was 64.0 min [50.5–80.5], and the average time of capnoperitoneum was 50.5 min [35.0–69.5]. The ventilation strategy resulted in mean (SD) plateau pressures of 24.2 (2.3) mbar, with tidal volumes of 517 (75.5) ml resulting in a respiratory system compliance of 37.9 (4.8) ml/mbar. Table 2 summarizes the perioperative anesthetic management.

**Table 1:** Baseline characteristics.

	**All patients**
	***N*** = **30**
Age (years) [median (IQR)]	43.0 [32.5; 48.0]
Gender (female) [*n* (%)]:	19 (63.3%)
Height (cm) [median (IQR)]	172 [160; 176]
Weight (kg) [median (IQR)]	130 [116; 146]
Body mass index (BMI; kg.m^−2^) [median (IQR)]	45.6 [42.4; 49.7]
**ASA physical status** [*n* (%)]:	
II	4 (13.3%)
III	25 (83.3%)
IV	1 (3.33%)

IQR, Interquartile range.

**Table 2:** Perioperative anesthetic management.

**Induction of anesthesia**	
Propofol [*n* (%)]:	30/30 (100%)
Total Propofol (mg) [median (IQR)]	200 [200; 240]
Succinylcholine	23/30 (76.7%)
Total Succinylcholine (mg) [median (IQR)]	150 [138; 195]
Rocuronium	13/30 (43.3%)
Total Rocuronium (mg) [median (IQR)]	70.0 [30.0; 90.0]
Fentanyl	30/30 (100%)
Total Fentanyl (mcg) [median (IQR)]	200 [150; 200]
Remifentanil	1/30 (3.3%)
Total Remifentanil (mcg)	150
Ketamine	4/30 (13.3%)
Ketamine 20 mg [*n* (%)]:	1/4 (25.0%)
Ketamine 30 mg [*n* (%)]:	1/4 (25.0%)
Ketamine 50 mg [*n* (%)]:	2/4 (50.0%)
Regional anesthesia with Ropivacaine [*n* (%)]:	30/30 (100%)
Total Ropivacaine 0.375% (mg) [median (IQR)]	150 [150; 150]
**Maintenance of anesthesia**	
Sevoflurane [*n* (%)]:	28/30 (93.3%)
Desflurane [*n* (%)]:	2/30 (6.7%)
MAC [median (IQR)]	0.80 [0.70; 0.80]
Dexmedetomidine	29/30 (96.7%)
Total Dexmedetomidine (mcg) [median (IQR)]	85.3 [64.0; 94.0]
Ketamine	19/30 (63.3%)
Total Ketamine (mg) [median (IQR)]	35.0 [30.0; 40.0]
Rocuronium	29/30 (96.7%)
Total Rocuronium (mg) [median (IQR)]	60.0 [40.0; 70.0]
Fentanyl	27/30 (90.0%)
Total Fentanyl (mcg) [median (IQR)]	150 [100; 225]
Remifentanil	6/30 (20.0%)
Total Remifentanil (mcg) [median (IQR)]	455 [302; 634] [*N* = 3]
**Procedure-related durations and oxygen desaturation**	
Duration of anesthesia (min) [median (IQR)]	149 [123; 168]
Duration of surgery (min) [median (IQR)]	64.0 [50.5; 80.5]
Duration of capnoperitoneum (min) [median (IQR)]	50.5 [35.0; 69.5]
**Type of laparoscopic bariatric surgery:**	
Gastric sleeve [*n* (%)]:	24/30 (80.0%)
Gastric bypass [*n* (%)]:	6/30 (20.0%)
Desaturation (SpO_2_ <90%) [*n* (%)]:	6/30 (20.0%)

### Primary outcome

**Figure 2:**
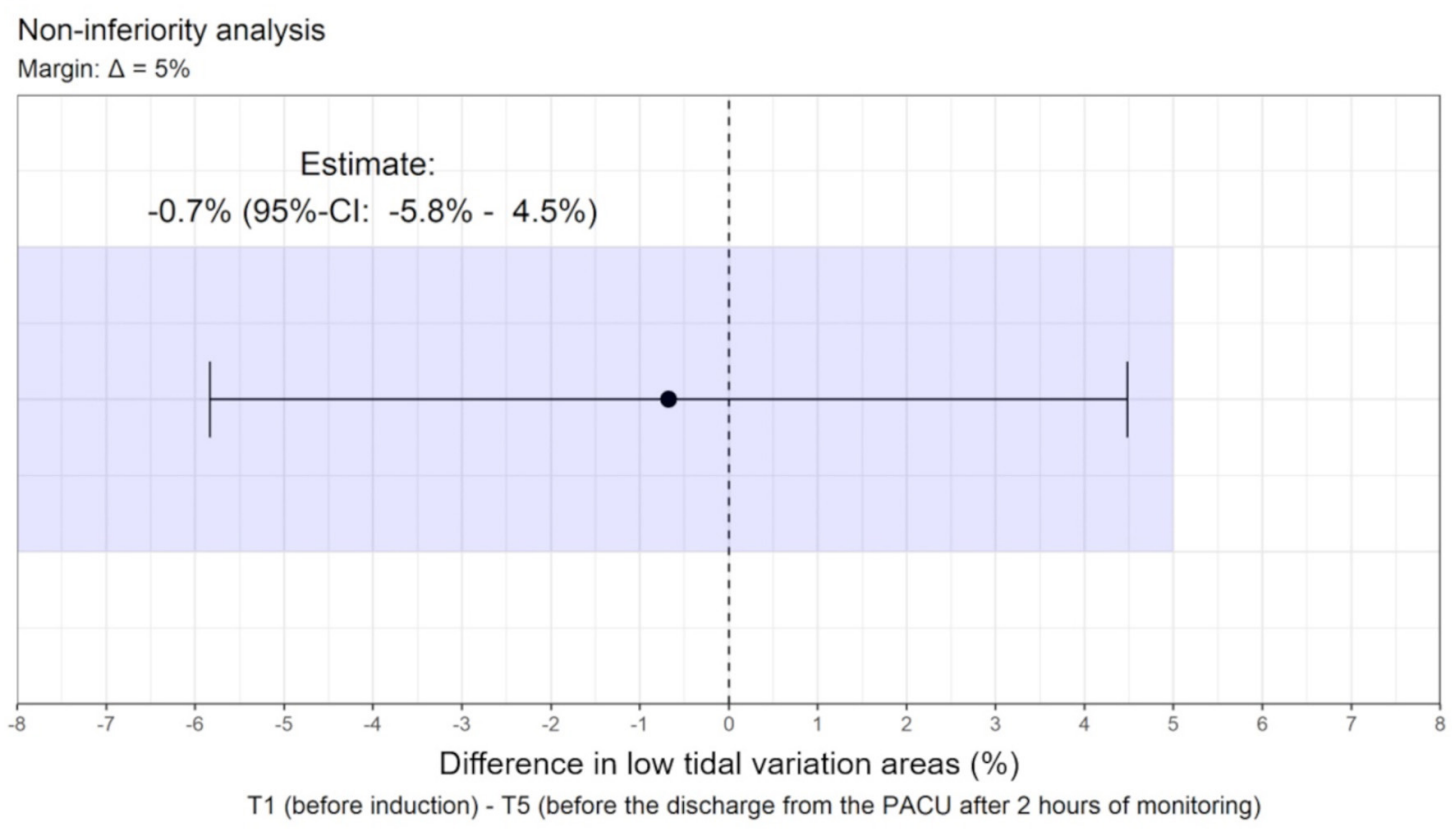
The mean difference in the percentage of low tidal variation areas between T1 and T5 and non-inferiority margin.

The mean difference in the percentage of low tidal variation areas between T5 and T1 was −0.7% (95% CI: −5.8% −4.5%), i.e., less than the predefined non-inferiority margin of 5%. The *p*-value for non-inferiority was *p* = 0.022. Therefore, we can assume non-inferiority at T5 compared to T1 (Figure 2).

### Secondary outcomes

**Figure 3:**
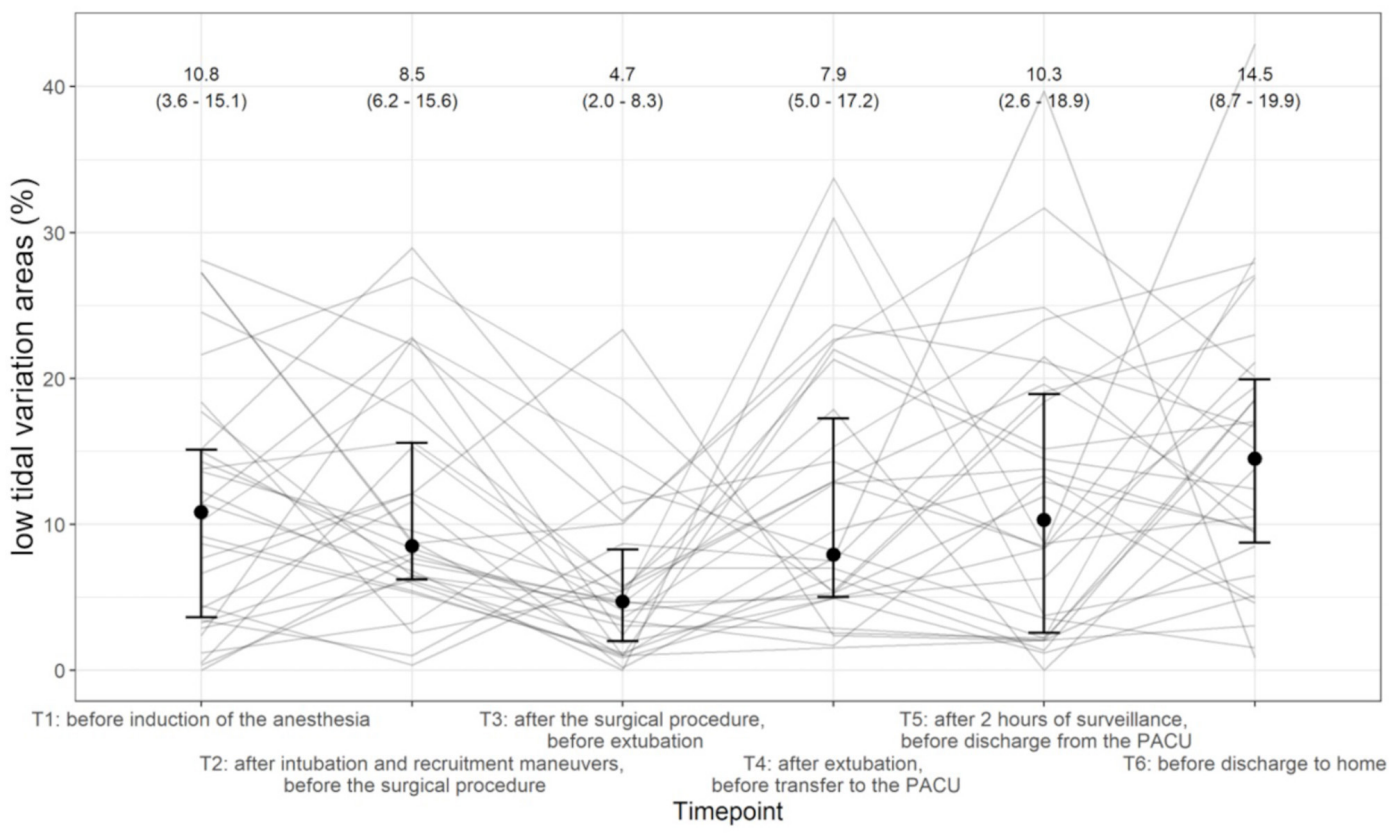
Evolution of proportion of low tidal variation areas across the different time points (T1 starting left until T6 on the right). Individual patients are shown as solid lines, and the median and interquartile range (IQR) at each time point are given.

**Figure 4:**
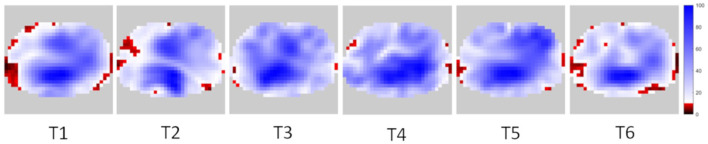
Example of distribution of low tidal variation areas (in red-black) at different time points.

The median (IQR) proportions of low tidal variation areas at the different time points were T1 10.8% [3.6–15.1%], T2 8.5% [6.2–15.6%], T3 4.7% [2.0–8.3%], T4 7.9% [5.0–17.2%], T5 10.3% [2.6–18.9%], and T6 14.5% [8.7–19.9%] (Figure 3). The proportion of low tidal variation areas differed over the six time points (*p* = 0.0006). All pairwise comparisons with respect to T3 showed significant differences. An example set of images is shown in Figure 4.

In an additional analysis, we demonstrate that one-third (nine subjects) of the subjects had a minimal (<5%) change, one-third (10 subjects) had more than a 5% increase, and one-third had more than a 5% decrease (11 subjects) of low tidal variation areas between T1 and T2. The group with the greatest reduction had significantly higher proportions of low tidal variation areas at baseline [median (IQR) 18.4% (15.0–27.3)] than the other two groups [median (IQR) 4.3% (0.5–11.4) and 7.7% (3.3–10.3); *p* < 0.001].

**Figure 5:**
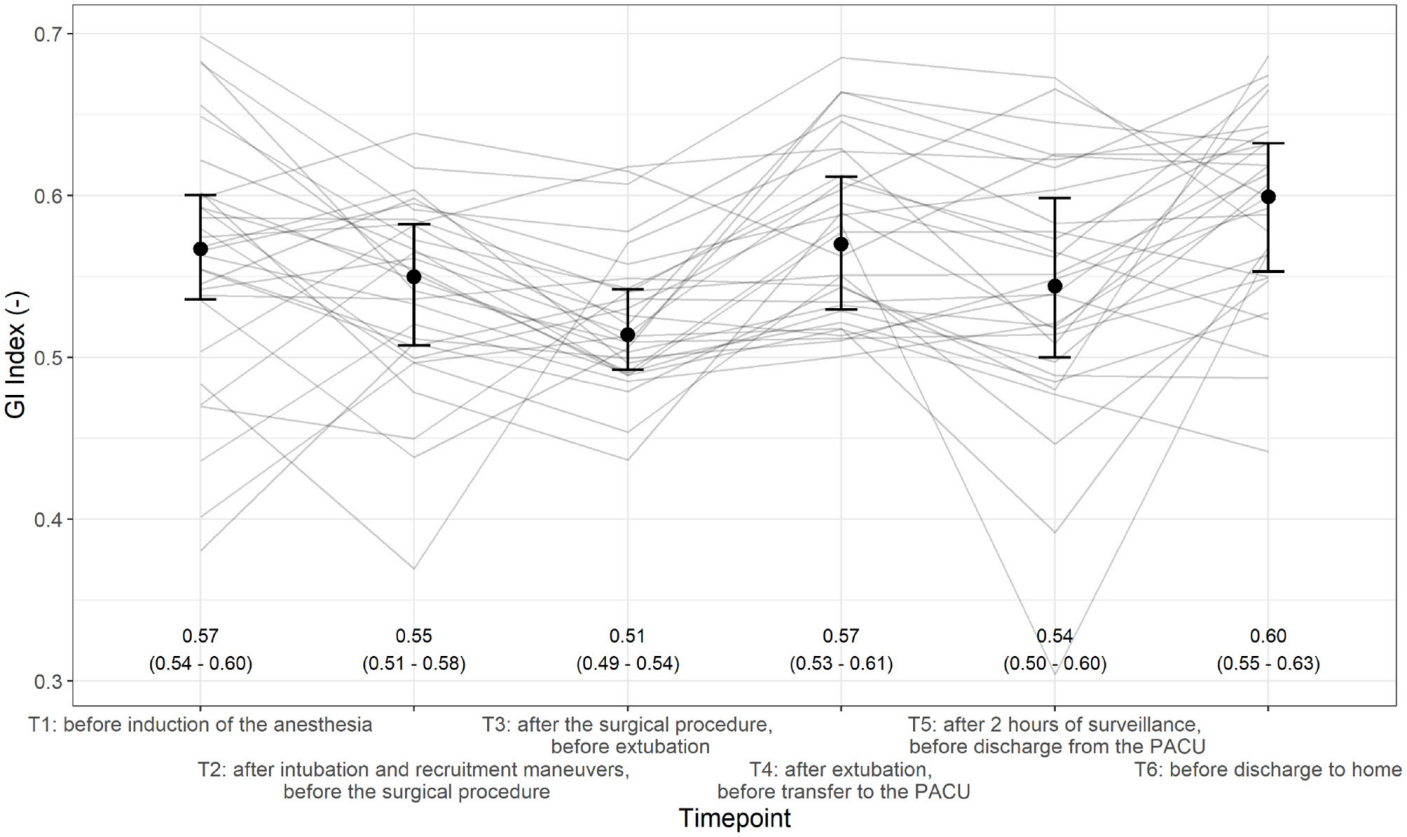
Evolution of the global inhomogeneity (GI) index across different time points (T1–T6). Individual patients are shown as solid lines, and the median and interquartile range (IQR) are given.

The mean [95% CI] GI indices at the different time points were T1 0.57 [0.54–0.60], T2 0.55 [0.51–0.58], T3 0.51 [0.49–0.54], T4 0.57 [0.53–0.61], T5 0.54 [0.50–0.60], and T6 0.60 [0.55–0.63] (Figure 5).

The proportion of GI indices differed over the six time points (*p* < 0.0001; Figure 3). All pairwise comparisons with respect to T3 showed significant differences. Additionally, we found significant differences between the following measurements: T2–T4 (*p* = 0.036) and T2–T6 (*p* = 0.007).

Six patients (20.0%) showed one [n = 4/6 (66.7%)] or two [n = 2/6 (33.3%)] episodes of desaturation SpO_2_ <90% intraoperatively during the surgical intervention. None of the patients reached any termination criteria during the procedure. Four patients showed one episode of desaturation SpO_2_ <90% during their PACU stay, which was treated with the administration of oxygen through a nasal cannula with a flow of 2–4 l/min.

None of the patients showed any postoperative pulmonary complications during the hospital stay. Seven patients (23.3%) required nasal oxygen with a flow of 1–3 l/min due to episodes of desaturation SpO_2_ <90% during the first 24 h. One patient showed thoracic pain limiting inspiration after discharge from PACU, and cardiac causes were excluded.

### Follow-up

All patients were at home during the telephone follow-up on day 14 after the surgery. Only one patient (pre-known asthma) showed mild postoperative pulmonary complications (more dyspnea than before the surgical procedure). Self-treatment with asthma medication occurred at home, with no medical presentation or re-hospitalization, not fulfilling the pre-defined criteria.

## Discussion

This prospective observational trial showed that obese patients undergoing laparoscopic bariatric surgery do not leave the PACU with increased low tidal variation areas as detected preoperatively. This finding suggests that perioperative formation of atelectasis might be successfully inhibited with a regimen of 10 cm H_2_O PEEP and repeated recruitment maneuvers.

The duration of laparoscopy and, thus, the increase in intra-abdominal pressure due to the pneumoperitoneum did not cause an increase in low tidal variation areas. On the contrary, after the termination of the pneumoperitoneum at T3 (after surgical procedure, before extubation), the GI index and the proportion of low tidal variation areas were significantly lower than at all other time points, suggesting the lowest rate of atelectasis and the most homogeneous ventilation during the entire perioperative period. This is particularly important, since in pneumoperitoneum, the dependent lung is expected to have increased rates of atelectasis (26). Our data indicate that an intraoperative ventilatory strategy with a volume-controlled mode with a tidal volume of 6–8 ml/kg, a PEEP of 10 cm H_2_O, and repeated recruitment maneuvers may prevent atelectasis formation. A second explanation might be the almost upright positioning during bariatric surgery to optimize laparoscopic surgical conditions.

Our data show that atelectasis formation can be successfully prevented perioperatively in bariatric patients undergoing one of the commonplace minor surgeries with a pneumoperitoneum duration of <1 h. Our study included patients with a BMI <40 with a median BMI of 45. These patients may benefit more from the ventilation strategy with a PEEP of 10 cm H_2_O than patients with less pronounced obesity.

Since Futier et al. (10) showed the benefits of using lung protective ventilation in abdominal surgery, several studies have been performed to shed light on the best ventilation strategies. In the large PROVHILO trial, which included non-bariatric patients, the authors suggested using low PEEP and no recruitment maneuvers in open abdominal surgery (27). The PROBESE collaborative group postulated, in the largest trial with obese patients, that postoperative pulmonary complications could not be reduced by elevated PEEP levels or recruitment maneuvers (28). Other studies showed that PEEP levels of 10 cm H_2_O with recruitment maneuvers improved respiratory parameters intraoperatively (29) and showed there was less postoperative atelectasis in the PACU (1, 29).

A published international consensus recommendation in 2019 (30) claimed that, although lung protective ventilation strategies could reduce postoperative pulmonary complications, there was still no consensus on its clinical use. Recommendations included individually titrated PEEP and performance of recruitment maneuvers with the lowest possible pressure over the shortest possible time, considered in an individual benefit–risk evaluation. Although this consensus does not solely pertain to bariatric patients, a BMI of >40 is regarded as one of the most significant risk factors for postoperative pulmonary complications, with an almost total agreement of experts (30). Nevertheless, it seems challenging to establish a direct relationship between intraoperative atelectasis and postoperative outcome (26).

Surprisingly, in the current study, this management not only prevented atelectasis formation but there was also no time point during the five EIT measures at which there was a significantly higher rate of low tidal variation areas than at baseline before administering hypnotics, oxygen, neuromuscular blocking agents, or opiates, thus matching the physiologic baseline of this patient group. This is surprising since reduced lung capacities and volumes during controlled ventilation and general anesthesia can lead to atelectasis in up to 90% of patients (31).

Different studies show that recruitment maneuvers and PEEP before extubation did not improve oxygenation in the PACU (32), an intraoperative regimen of increased PEEP level and recruitment maneuvers in bariatric patients did not reduce postoperative pulmonary complications (33), and PEEP and recruitment maneuvers for an open abdominal surgery cohort did not reduce postoperative pulmonary complications (27). In contrast, our data for this specific cohort of patients (BMI ≥ 40, laparoscopic surgery, pneumoperitoneum time <60 min, anti-Trendelenburg position) suggest an improvement regarding atelectasis formation due to the chosen anesthesia management (volume-controlled ventilation with 6–8 ml/kg, 10 cm H_2_O PEEP, FiO_2_ at 0.6, and recruitment maneuvers). However, what effect this ultimately has on postoperative pulmonary complications remains unclear.

These findings are consistent with data showing that recruitment maneuvers reduced pulmonary dysfunction in the PACU during laparoscopic bariatric surgeries (29) or that PEEP and recruitment maneuvers decreased atelectasis in bariatric patients (4). Recruitment maneuvers could lead to pulmonary conditions on a level comparable to preoperative conditions in obese patients (13). However, three standardized recruitment maneuvers were performed within a median of 64 min of the operation time, which does not often correspond to clinical reality, especially in prolonged procedures. In addition, patients are positioned in an anti-Trendelenburg position during laparoscopic bariatric procedures, which could reduce atelectasis compared with laparoscopic procedures in the supine position or the Trendelenburg position.

Limitations of this study include the single-center design and the lack of randomization with the absence of a control group (without PEEP of 10 cm H_2_O and recruitment maneuvers). Nevertheless, we demonstrated an absence of significant low tidal variation areas in this cohort. There are some more limitations in the measurement technique itself. First, the EIT belt was placed in the same position on the skin of the patients, but postural changes among measurements, muscle paralysis, and capnoperitoneum could all affect the position and geometry of the lungs inside the chest. However, the measurements for the primary outcome should not be affected because all measurements were performed under identical conditions. We cannot exclude an effect of the described phenomenon on our secondary outcomes. Second, we used a modified analysis algorithm for low tidal variation areas compared to the original publication describing the so-called silent spaces. The original technique, analyzing only pixels within anatomically (CT-derived) defined lung regions, carries the risk of underestimating minimally ventilated lung areas (21). This is the case when the assumed lung areas do not match the effective anatomy of the measured lung. Our analysis technique avoids a priori assumptions and takes into account all pixels of the reconstructed EIT image. This approach tends to overestimate the low tidal variation areas because areas outside the lung are also included. For the comparison of different time points in the same patient, we believe that the second analysis technique is better suited because, with this technique, all minimally ventilated lung segments are included in the analysis. The sensitivity and specificity of silent spaces measured by EIT are not known and its accuracy depends on the “right” assumptions regarding lung anatomy (33). Nevertheless, the relevance of this finding on repetitive measurements of low tidal variation areas in the same subject remains unclear. Additionally, there is evidence that the change in silent spaces correlates well with lung recruitment measured using the P-V curve technique (19).

## Conclusion

Obese patients undergoing laparoscopic bariatric surgery do not leave the PACU with an increased rate of low tidal variation areas as detected preoperatively. Thus, we suggest applying 10 cm H_2_O PEEP and repeated recruitment maneuvers perioperatively to potentially prevent atelectasis formation.

## Data availability statement

The raw data supporting the conclusions of this article will be made available by the authors, without undue reservation.

## Ethics statement

The studies involving humans were approved by Ethikkommission für die Forschung am Menschen, Bern. The studies were conducted in accordance with the local legislation and institutional requirements. The participants provided their written informed consent to participate in this study.

## Author contributions

MB, LR, MK, ML, TRiv, and AV contributed to the study design, study conduct, analysis, manuscript preparation, patient recruitment, and manuscript finalization. AF contributed to the study design, study conduct, analysis, and manuscript preparation. MH helped with the statistical analysis. TRie contributed to the study design, study conduct, analysis, manuscript preparation, statistical analysis, and finalizing the manuscript. All authors contributed to the article and approved the submitted version.
